# Integrative dissection of 5-hydroxytryptamine receptors-related signature in the prognosis and immune microenvironment of breast cancer

**DOI:** 10.3389/fonc.2023.1147189

**Published:** 2023-09-19

**Authors:** Dandan Zhan, Xuan Wang, Yifeng Zheng, Shengqi Wang, Bowen Yang, Bo Pan, Neng Wang, Zhiyu Wang

**Affiliations:** ^1^ State Key Laboratory of Dampness Syndrome of Chinese Medicine, The Second Affiliated Hospital of Guangzhou University of Chinese Medicine, Guangzhou, China; ^2^ Guangdong-Hong Kong-Macau Joint Lab on Chinese Medicine and Immune Disease Research, Guangzhou University of Chinese Medicine, Guangzhou, Guangdong, China; ^3^ Guangdong Provincial Key Laboratory of Clinical Research on Traditional Chinese Medicine Syndrome, Guangdong Provincial Academy of Chinese Medical Sciences, Guangdong Provincial Hospital of Chinese Medicine, Guangzhou, Guangdong, China; ^4^ Integrative Medicine Research Center, School of Basic Medical Sciences, Guangzhou University of Chinese Medicine, Guangzhou, Guangdong, China

**Keywords:** breast cancer, depression, risk signature, 5-hydroxytryptamine receptors, cancer immune microenvironment

## Abstract

**Background:**

Depression increases the risk of breast cancer recurrence and metastasis. However, there lacks potential biomarkers for predicting prognosis in breast cancer. 5-hydroxytryptamine (5-HT) plays a key role in the pathogenesis and treatment of depression. In this study, we developed a prognostic signature based on 5-HT receptors (5-HTRs) and elucidated its potential immune regulatory mechanisms for breast cancer prognosis.

**Methods:**

Oncomine, GEPIA, UALCAN, cBioPortal, Kaplan-Meier plotter, and TIMER were used to analyze differential expression, prognostic value, genetic alteration, and immune cell infiltration of HTRs in breast cancer patients. The model training and validation assays were based on the analyses of GSE1456 and GSE86166. A risk signature was established by univariate and multivariate Cox regression analyses. The transwell assay was utilized to verify the effect of the 5-HTRs expression on breast cancer invasion. Effects of HTR2A/2B inhibitor on CD8^+^ T cell proliferation and infiltration as well as apoptosis of 4T1 cells in the tumor microenvironment were detected by flow cytometry and TUNEL assay. Zebrafish and mouse breast cancer xenografts were used to determine the effect of HTR2A/2B inhibitor on breast cancer metastasis.

**Results:**

The expression levels of HTR1A, HTR1B, HTR2A, HTR2B, HTR2C, HTR4, and HTR7 were significantly downregulated in highly malignant breast cancer types. 5-HTRs were significantly associated with recurrence-free survival (RFS) in breast cancer patients. The genetic alteration of HTR1D, HTR3A, HTR3B, and HTR6 in breast cancer patients was significantly associated with shorter overall survival (OS). Finally, HTR2A and HTR2B were determined to construct the risk signature. The expression of HTR2A/2B was positively correlated with the infiltration of immune cells such as CD8^+^ T cells and macrophages. Furthermore, inhibition of HTR2A expression could suppress CD8^+^ T cell proliferation and enhance invasion and metastasis of breast cancer cells in both zebrafish and mice model.

**Conclusions:**

The HTR2A/2B risk signature not only highlights the significance of HTRs in breast cancer prognosis by modulating cancer immune microenvironment, but also provides a novel gene-testing tool for early prevention of depression in breast cancer patients and lead to an improved prognosis and quality of life.

## 1 Introduction

As reported in the World Health Statistics 2020, breast cancer is now the leading malignancy in women, which accounted for 11.7% of new cases and 6.9% of deaths worldwide in 2020 (1, 2). Depression is a common co-morbidity of breast cancer with a global prevalence of 32.2% (3). A meta-analysis showed that depression was associated with breast cancer recurrence, all-cause mortality, and tumor-specific mortality as independent predictors of breast cancer recurrence and survival (4). A number of animal studies have also shown that chronic psychological stress promotes the growth and metastasis of breast cancer in mice (5–7). These studies highlight the close association between depression and breast cancer prognosis. Therefore, alleviating depression in breast cancer patients is clinically valuable and necessary.

Clinical practice guidelines recommend meditation, relaxation, yoga, massage, and music therapy for breast cancer patients with depression or mood disorders (8, 9). Importantly, anti-depression treatment could improve their quality of life and possibly extend the longevity (10). Although these therapies can partially improve depression, effective precision therapies are lacking. Currently, the main patient-reported depression measures in cancer include the Hospital Anxiety Depression Scale (HADS), the Beck Depression Inventory (BDI), and the Center for Epidemiologic Studies Depression Scale (CES-D), but they all have limitations (11). Therefore, it is urgent to find effective biomarkers to predict depression in breast cancer and provide new strategies for targeting treatment.

5-hydroxytryptamine (5-HT) neurotransmission plays a key role in the pathogenesis and treatment of depression (12). Notably, the effects of 5-HT (or serotonin) are mediated by serotonin receptors and serotonin transporters (SERT), and at least 15 different 5-HTR subtypes have been identified. Besides 5-HTR3, the majority of 5-HTRs belong to the G-protein-coupled receptor superfamily (GPCRs) (13). Several studies have revealed that different HTRs are overexpressed in different cancers, and serotonin could promote cancer growth by activating serotonin receptors on cancer cells (13, 14). However, some studies have reported that the expression of 5-HTR1B was low in melanoma and gastrointestinal stromal tumor cells, as well as in endothelial cells of the colon, ovary, breast, kidney, and pancreatic malignancies (15). In addition, a significant decrease in HTR1E expression was found in peritoneally disseminated ovarian cancer cells (16). Currently, there lacks a comprehensive understanding of different HTR expression levels, genetic variants, and their associations with prognosis in breast cancer patients.

It is known that serotonin and 5-HTRs in the tumor microenvironment could affect cancer growth through the crosstalk between immune cells and the nervous system. A significant correlation has been found between blood serotonin levels and the inflammatory state of the body, as reflected in the balance of Th1/Th2 cells (17). Compared to CD4^+^ helper T cells, CD8^+^ Cytotoxic T Lymphocytes (CD8^+^ CTLs) are more likely to express serotonergic components such as recombinant tryptophan hydroxylase 1 (TPH1), monoamine oxidase type-A (MAO-A), and vesicular monoamine transporter 1 (VMAT1) (18, 19). The expression of these enzymes increases further upon activation, and T cells actively respond to the autocrine-produced serotonin (18). Meanwhile, activated T cells could activate 5-HTR7, 5-HTR1B, 5-HTR2A, and synthesize 5-HT autocrinely (20). Furthermore, the proliferation of human and murine Th1 cells, as well as cytotoxic CD8^+^ T cells, was promoted by 5-HTR1B and 5-HTR2A. The selective 5-HTR1B antagonist SB-216641 and the 5-HTR2A inhibitor sarpogrelate hydrochloride were found to inhibit IL-2 and IFN-γ produced by T cells (21, 22). 5-HT1A receptor-mediated cell survival and S-phase transition in T cells were also shown to be associated with NF-κB translocation into the nucleus (23). All these findings suggest that 5HTRs may affect cancer prognosis *via* modulating immunity. However, the molecular mechanisms by which serotonin alters the phenotype and function of immune cells have not been well explored.

Herein, we systematically analyzed the expression and prognostic value of different HTRs in breast cancer and established a risk signature, as well as explored its relation with the activities of immune cells. We found that most HTRs expressions were suppressed in highly malignant breast cancer, and all of them were significantly associated with recurrence-free survival (RFS). HTR2A and HTR2B were identified to establish a new risk signature, which was significantly correlated with the infiltration of immune cells and affected cancer metastasis by *in vivo* experimental validation. In summary, our study not only provides a novel risk signature for prognosis prediction of breast cancer based on HTRs, but also highlights the activation of HTR2A as a novel therapeutic strategy to improve breast cancer prognosis *via* mobilizing CD8^+^T cells.

## 2 Materials and methods

### 2.1 Transcriptional levels of HTRs in patients with breast cancer

The mRNA levels of different HTRs in diverse cancer types were analyzed in Oncomine (https://www.oncomine.org/resource/login.html), a publicly accessible online database for analyzing cancer microarray information (24). The HTRs mRNA with a *p*-value< 0.05, a fold change (FC) of 2 was considered as significant.

### 2.2 GEPIA dataset analysis

Gene Expression Profiling Interactive Analysis (GEPIA, http://gepia.cancer-pku.cn/index.html) is capable of analyzing RNA sequencing data from thousands of cancer and normal samples using standard pipelines (25). Through this web server, the association between HTRs mRNA expression levels and breast cancer stages was studied by GEPIA, and Student’s *t*-test was used to test the significance. We used “major stage” for plotting and “log_2_ (TPM + 1)” for log-scale.

### 2.3 cBioPortal and UALCAN analysis

cBioPortal (www.cbioportal.org) is a comprehensive web resource that provides cancer genomics data (26), from which genetic alterations and the network module of HTRs were obtained. In our study, the genome atlas consisted of 960 complete samples with mutation, CNA and expression data. The genetic alterations of HTRs were analyzed by the following parameters: putative copy number alteration (CNA) data from GISTIC, z-values of mRNA expression relative to all samples (Log RNA Seq V2 RSEM), and mutations. The expression levels of 15 HTRs based on major subclasses were analyzed on UALCAN (https://ualcan.path.uab.edu/analysis.html) web sourced from the TCGA database.

### 2.4 The Kaplan–Meier analysis

Kaplan-Meier analysis (http://kmplot.com/analysis/) was applied to evaluate the prognostic value of HTRs mRNA expression in breast cancer patients. In the database, the Kaplan-Meier survival curve was generated to validate the distribution of survival time based on the optimal cutoff values of HTRs mRNA expression. The number of patients, median value of mRNA expression, 95% CIs, HRs, and *p*-values were all provided following the analysis.

### 2.5 TIMER analysis

TIMER (www.cistrome.shinyapps.io) is a user-friendly online analysis tool that can evaluate different tumor-infiltrating immune cells and their clinical impact (27). In the present study, TIMER was used to analyze the association between the expression level of HTRs and immune cell infiltration.

### 2.6 Identification of prognostic genes and construction of a risk model

In the model training cohort of GSE1456 with 159 breast cancer patients, the correlation between the genes in the dataset and RFS was carried out by univariate Cox analysis. The HTRs with *p*< 0.05 were identified as candidate genes. Multivariate Cox analysis was subsequently applied to screen the highly prognosis-associated genes, and a prognostic risk model was constructed according to the following formula:


$$\text{Risk Score}(\text{patient})=\sum_{i=1}^{n}\beta \text{i}Exp\text{i}.$$

(where “Exp” represents gene expression level and “β” represents the regression coefficient from the multivariate Cox analysis).

### 2.7 Validation of the risk signature

The cohort of GSE86166 was utilized as the validation dataset, in which 156 breast cancer patients were included. Kaplan-Meier survival analysis along with log rank test were performed to analyze the survival difference between patients with different scores.

### 2.8 Cell culture

4T1 murine breast cancer cells were purchased from the American Type Culture Collection (ATCC) and cultured in DMEM complete medium. E0771 murine breast cancer cells were obtained from Jennio Biotech Co.,Ltd (GuangZhou, China) and cultured in RPMI-1640 medium supplemented with 10% FBS and 1% PS. RPMI-1640 complete medium was used to culture CD8^+^ T cells isolated from the spleen of female Balb/c mice. Cells were maintained in a humidified incubator with 5% CO_2_ at 37°C.

### 2.9 Flow cytometry assay and CFSE staining assay

The FACSAria III flow cytometer (BD Biosciences, California, US) or the NovoCyte Quanteon Flow cytometer (Agilent Technologies, California, USA) was used to analyze and sort the population of CD8^+^ T cells. The antibodies used in the flow cytometry assay were APC - conjugated CD8a antibody (100711, Biolegend, California, US) and FITC-conjugated CD3 antibody (100204, Biolegend, California, US). The sorted CD8^+^ T cells were re-suspended in RPMI-1640 complete culture medium and stimulated by 50 ng/ml phorbol 12-myristate 13-acetate (PMA), 1 ug/ml ionomycin, and 10 ng/ml IL-2 (Multiscience, Hangzhou, China) *in vitro* for 48 h, and then continuously cultured in RPMI-1640 complete culture medium.

To assess the effects of HTR2A or HTR2B inhibitor (ketanserin or RS-127445 hydrochloride) on the proliferation of CD8^+^ T cells, 5 (6)-Carboxyfluorescein diacetate succinimidyl ester (CFSE) staining assay was performed by CFSE (C1031, Beyotime, Shanghai, China) for 15 min. Subsequently, cells were re-suspended with RPMI-1640 medium and seeded in the 96 well plates. After treatment with 0.1 nM ketanserin (HTR2A inhibitor, 74050-98-9, MCE) or 0.1 nM RS-127445 hydrochloride (HTR2B inhibitor,199864-86-3, MCE) for 24 h, CD8^+^ T cells were collected and analyzed by the NovoCyte Quanteon Flow cytometer.

### 2.10 Cell apoptosis analysis

Cell apoptosis was evaluated by flow cytometry analysis. Firstly, CD8^+^ T cells were treated with HTR2A inhibitor ketanserin or HTR2B inhibitor RS-127445 hydrochloride for 24 h as described above. Next, we co-cultured the pretreated-CD8^+^ T cells with 4T1 (or E0771) cells in RPMI-1640 complete culture medium at a ratio of 5:1 for 24 h, and the attached 4T1 cells or E0771 cells were subsequently harvested. Then, cells were plated at a density of 3 × 10^5^ cells per well in six-well plates overnight. Annexin V-FITC/PI staining was performed according to the manufacturer’s instruction (Multi Sciences, Hangzhou, China), and analyzed by the NovoCyte Quanteon Flow cytometer.

### 2.11 Transwell assay

Firstly, 4T1 cells or E0771 cells co-cultured with the pretreated-CD8^+^ T cells were obtained according to the above method. Then, for the transwell assay, 100 μl matrigel (200 μg/mL, 354248, Corning, NY, USA) was smoothly spread on the bottom chamber (8 μm pore size, Millipore, Billerica, MA, USA) of the 24-well transwell insert to simulate the basement membrane, and incubated for 3 h at 37°C for gelling. Following that, 4T1 cells or E0771 cells were seeded in the upper chamber, and the bottom chamber was filled with the complete culture medium. After 24 h, the non-invaded cells were cleared from the upper chamber, and 0.1% coomassie blue was used to stain invaded cells on the bottom of the upper chamber.

### 2.12 Zebrafish breast cancer xenotransplantation model

To examine the effect of HTR2A/2B inhibitor on breast cancer metastasis *in vivo*, a zebrafish xenograft of breast cancer was established. Briefly, 5 μM 1, 1’-Dioctadecyl-3,3,3’,3’ -tetramethylindocarbocyanine perchlorate (Dil, 281 Sigma-Aldrich) perchlorate was used to stain 4T1 cells or E0771 cells. The sorted and stimulated CD8^+^ T cells were treated with ketanserin or RS-127445 hydrochloride for 24 h. Dil-stained 4T1 cells or E0771 cells and pretreated-CD8^+^ T cells at a ratio of 1:5 were injected into each zebrafish embryo with a microsyringe at 48 h post fertilization. An inverted fluorescence microscope (Nikon Eclipse C1, Tokyo, Japan) was used to monitor the growth and metastasis of 4T1 cells or E0771 cells after 48 h.

### 2.13 Animal experiments

All animals feeding and experimental treatment were approved by the Guangdong Provincial Hospital of Chinese Medicine Animal Ethics Committee (No. 2023075). Four-week-old female Balb/c mice were obtained from the Guangdong Experimental Animal Center (Guangzhou, China), raised under specific pathogen-free conditions at the Experimental Animal Center of Guangdong Provincial Hospital of Chinese Medicine, and given sterilized food and water. To generate the 4T1-Luc breast cancer model, 2×10^6^ 4T1-Luc cells were re-suspended in 200 μl PBS and inoculated subcutaneously into the mammary fat pads of mice. When the tumor volume reached approximately 100 mm^3^, the mice were randomly divided into three groups: vehicle control group, IL-2 treated CD8^+^T cells group and HTR2A inhibitor treated CD8^+^T cells group (n=6). Firstly, CD8^+^ T cells were treated with IL-2 or HTR2A inhibitor for 24 h as described above. Next, the pretreated-CD8^+^ T cells at a density of 5 × 10^6^ were injected into the tail veins of mice every 2 weeks. To monitor the tumor growth, tumor volume was measured every 3 days. At the end of treatment, the mice were intraperitoneally injected with D-Luciferin (150 mg/kg, PerkinElmer, Boston, United States) and photographed with the IVIS imaging system (IVIS-spectrum, Perkin Elmer, Boston, United States). When the tumors reached 1.5 cm in diameter, the mice were euthanized, and the lungs and tumors were removed and photographed.

### 2.14 HE staining assay and TUNEL assay

Paraffin-embedded sections were treated twice with xylene for 10 min each, then rehydrated with 100% to 70% ethanol, followed by 10% hematoxylin to show the nuclei and 1% eosin to stain the cytoplasm. Finally, the specimens were dehydrated, cleaned, and sealed before observation under the BX61 microscope (Olympus, Center Valley, PA, USA). The TUNEL assay was conducted using the TUNEL Kit (C1088, Beyotime, Shanghai, China) according to the manufacturer’s instructions. Briefly, paraffin sections of tumor tissue were dewaxed and processed with proteinase K to strip proteins from the nuclei. Sections were incubated with terminal deoxynucleotidyl transferase at 37°C for 1 h, followed by incubation with streptavidin-FITC in a humid chamber at 37°C for 30 min. Finally, fluorescence intensity was observed with a laser confocal microscope (LMS710, ZEISS, Jena, Germany) at excitation wavelengths of 450-500 nm and emission wavelengths of 515-565 nm.

### 2.15 Immunofluorescence

For immunofluorescence analysis, the tumor sections were cut (4 um thick), dewaxed, hydrated, and subjected to antigen retrieval by incubating slides in the boiled citrate buffer (0.01 M, pH 6.0). Next, tissue sections were permeabilized with 0.25% Triton X-100 for 20 min and then closed with 5% BSA for 30 min. Sections were then incubated overnight at 4°C with antibodies containing mouse anti-CD8 (14-0081-82, eBioscience, San Diego, CA, USA) and rabbit anti-HTR2A (DF8900, Affinity, Cincinnati, OH, United States), followed by incubation with 1:200 dilution of Alexa Fluor^®^555 conjugated-anti-mouse IgG (A21422, Invitrogen, Carlsbad, CA, United States) and Alexa Fluor^®^ 488 conjugated-anti-rabbit IgG (4412S, CST, Danvers, MA, United States) at room temperature for 1 h. Sections were then incubated with 4, 6-diamidino-2-phenylindole (DAPI, C1005, Beyotime, Shanghai, China) for nuclear staining. The final fluorescence images were taken using the LSM710 confocal microscope.

### 2.16 Statistical analysis

We analyzed the statistical data using R software (version 3.6.3) and plotted the Kaplan-Meier survival curves at 1, 3, and 5 years with the survminer package. Multivariate Cox analysis and univariate Cox analysis were used to assess the prognostic influence of the risk model. For the experiments, SPSS 26.0 was used to calculate statistics, data were presented as mean ± standard deviation (SD), the one-way ANOVA and the Dunnett’s or Bonferroni *post hoc* test were performed to compare multiple groups. *p*< 0.05 was considered statistically significant.

## 3 Results

### 3.1 Transcriptional expression of HTRs in breast cancer patients

**Figure 1:**
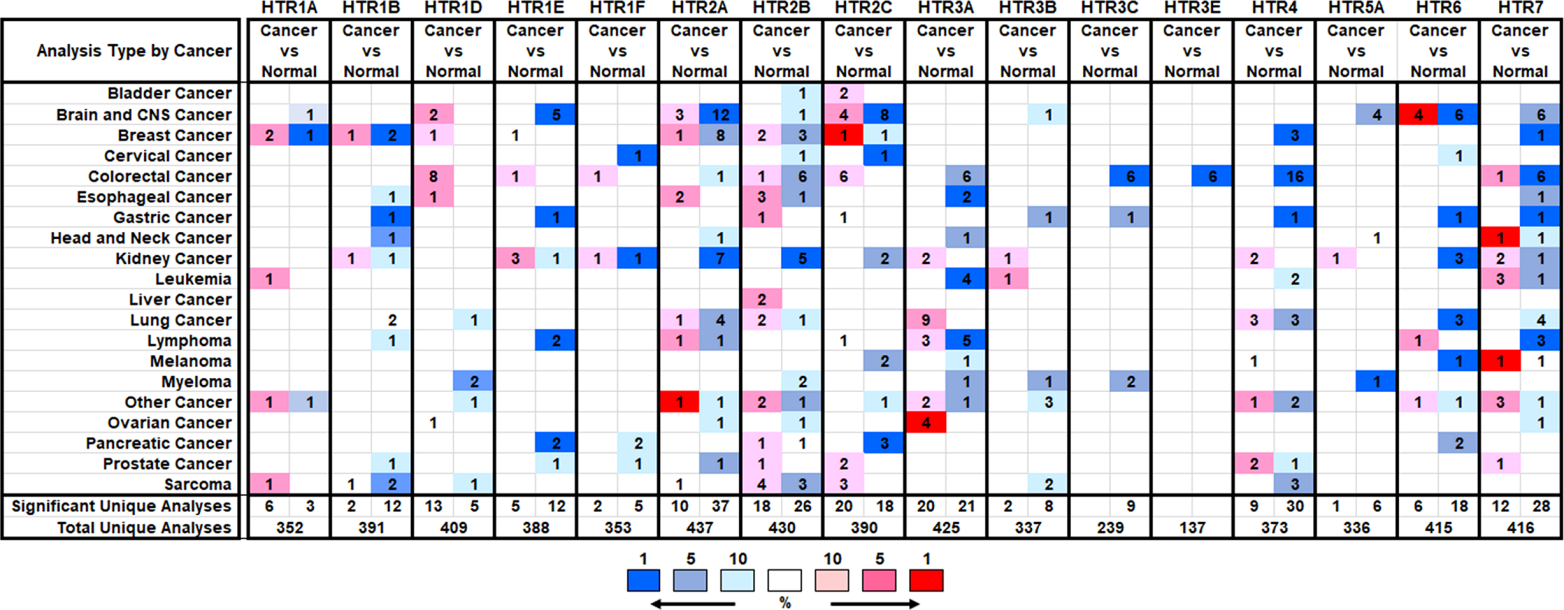
The Transcription expression of HTRs in Different Types of Cancers (Oncomine). The numbers of datasets with statistically significant alterations (*p*< 0.05, FC > 2) in the mRNA expression of HTRs were presented: upregulation (red) and downregulation (blue).

To explore the expression of HTRs in cancer tissues, we compared the mRNA expression of HTRs in different cancer and normal tissues using the Oncomine database. Sixteen HTRs were widely present in mammalian cells but are aberrantly expressed in different tumor tissues (**Figure 1**). The Oncomine database showed that the mRNA expression levels of different 5-HTR subtypes were mostly explored in the kidney, brain and CNS (central nervous system), breast, and gastric cancers, with 12, 10, 9, and 9 kinds of 5-HTRs being studied, respectively. Among these 5-HTRs, HTR7, HTR2A, HTR2B, and HTR2C were extensively studied in different tumor tissues.

The mRNA expression levels of 5-HTRs in breast cancer tissue are distinct in different databases (**Supplementary Table 1**). In two datasets, the mRNA expression levels of HTR1A were significantly upregulated in invasive lobular breast carcinoma and invasive breast carcinoma stroma. However, Gluck et al. showed that HTR1A was down-expressed in invasive breast carcinoma versus normal tissue with a FC of 4.533. In Turashvili Breast Statistics, HTR1B was overexpressed in invasive lobular breast carcinoma with a FC of 2.181, while it was downregulated in Radvanyi Breast Statistics. In the Cancer Genome Atlas (TCGA) breast statistics, HTR1D was found to be upregulated in intraductal cribriform breast adenocarcinoma (FC = 2.373), while HTR1E level was increased in mixed lobular and ductal breast carcinoma (FC = 2.082). With regard to the expression level of HTR2A, except for its overexpression in fibroadenoma (FC = 2.075, the Sorlie Breast Statistics), it was downregulated in male breast carcinoma (FC = -3.289), invasive breast carcinoma (FC = -3.808), mixed lobular and ductal breast carcinoma (FC = -3.484), intraductal cribriform breast adenocarcinoma (FC = -3.675), invasive lobular breast carcinoma (FC = -3.063), invasive ductal breast carcinoma (FC = -3.963), invasive ductal and lobular carcinoma (FC = -3.972) and mucinous breast carcinoma (FC = -4.763, TCGA breast statistics).

HTR2B was found overexpressed in ductal breast carcinoma *in situ* stroma (FC = 2.185) and invasive breast carcinoma stroma compared to normal samples (FC = 3.648). Nevertheless, it was showed that HTR2B was downregulated in invasive ductal breast carcinoma with a FC of 2.567 (The Radvanyi Breast Statistics), intraductal cribriform breast adenocarcinoma with a FC of 4.579 and mucinous breast carcinoma with a FC of 2.455 (TCGA breast statistics). HTR2C was found highly expressed in invasive lobular breast carcinoma with a FC of 2.517 (Turashvili Breast Statistics) and decreased in male breast carcinoma with a FC of 2.041 (TCGA breast statistics). HTR4 was decreased in mucinous breast carcinoma with a FC of 2.645, male breast carcinoma with a FC of 2.237 (TCGA breast statistics) and invasive ductal breast carcinoma stroma with a FC of 6.745 (The Karnoub Breast Statistics). In Radvanyi Breast Statistics, HTR7 was also found inhibited in invasive mixed breast carcinoma (FC = -2.548). In addition, Oncomine analysis showed no significant difference in the transcriptional levels of HTR1F, HTR3, HTR5 and HTR6 between breast adenocarcinoma and normal samples. Taken together, most HTRs expression are decreased in high-malignant breast cancer, but are upregulated in low-malignant breast cancer compared to normal breast tissue.

### 3.2 Relationship between the mRNA levels of HTRs and the clinicopathological parameters of patients with breast cancer

**Figure 2:**
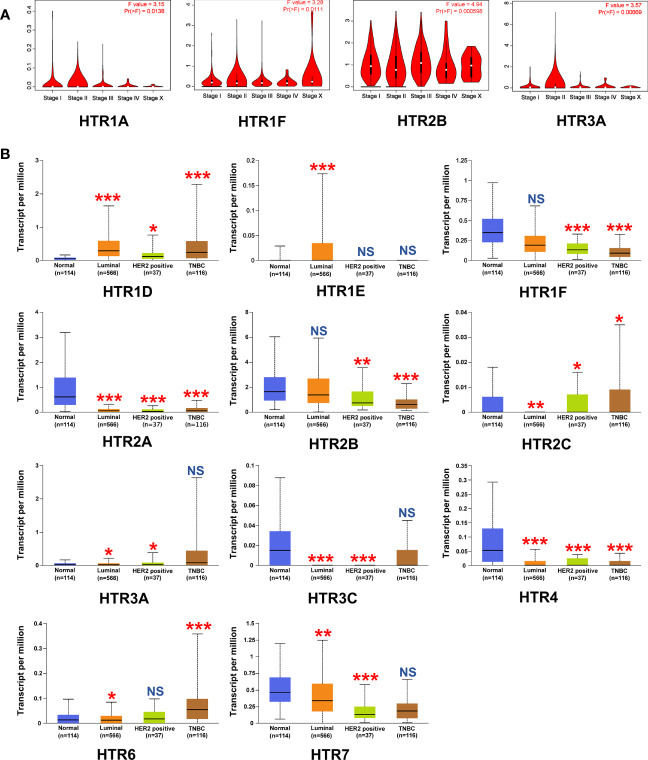
Relationship between the mRNA Levels of HTRs and the clinicopathological parameters of breast cancer patients. **(A)** Correlations between HTRs expression and tumor stage in breast cancer patients (GEPIA). **(B)** Expression of HTRs in different breast cancer subtypes from UALCAN database. *p< 0.05, **p< 0.01, ***p< 0.001, and NS (not significant) *vs.* the normal group.

Given that the mRNA levels of HTRs are significantly related to the malignancy in breast cancer patients, we were interested in further exploring whether there is a correlation between the levels of HTRs and breast tumor stage and pathological type. Thus, we used the GEPIA dataset to analyze the relationship between mRNA expression levels of HTRs and tumor stage in breast cancer patients. The results demonstrated that HTR1A/1F/2B/3A expression were varied significantly among different stages (**Figure 2A**), whereas the levels of HTR1B/1D/1E/2A/2C/3B/3C/4/5A/6/7 did not significantly differ (**Supplementary Figure 1A**).

To better identify the correlation between the HTRs expression levels and the clinicopathological features of breast cancer, we further analyzed the relationship between the HTRs expression and breast cancer subtypes by using the TCGA dataset. In the luminal breast cancer subtype, the results showed increases in the mRNA level of HTR1D/1E, decreases in HTR2A/2C/3A/3C/4/6/7, and no significant change in HTR1F/2B. As for HER2-positive breast cancer subtypes, the mRNA levels of HTR1D/2C/3A were upregulated, while the mRNA levels of HTR1F/2A/2B/3C/4/7 were downregulated, and no significant changes were found in HTR1E/6. With regard to triple-negative breast cancer (TNBC), the transcript levels of HTR1D/2C/6 were increased, HTR1F/2A/2B/4 expressions were decreased, and HTR1E/3A/3C/7 levels were unchanged (**Figure 2B**). In addition, the mRNA levels of HTR1B were not significantly changed in all breast cancer subtypes compared with normal breast tissue, and the mRNA for HTR1A/3B/5A were barely expressed (**Supplementary Figure 1B**). More importantly, we found that the expression of HTR1F and HTR2B were decreased with deterioration of pathology.

### 3.3 Prognostic value of HTRs in predicting the recurrence-free survival of breast cancer patients

**Figure 3:**
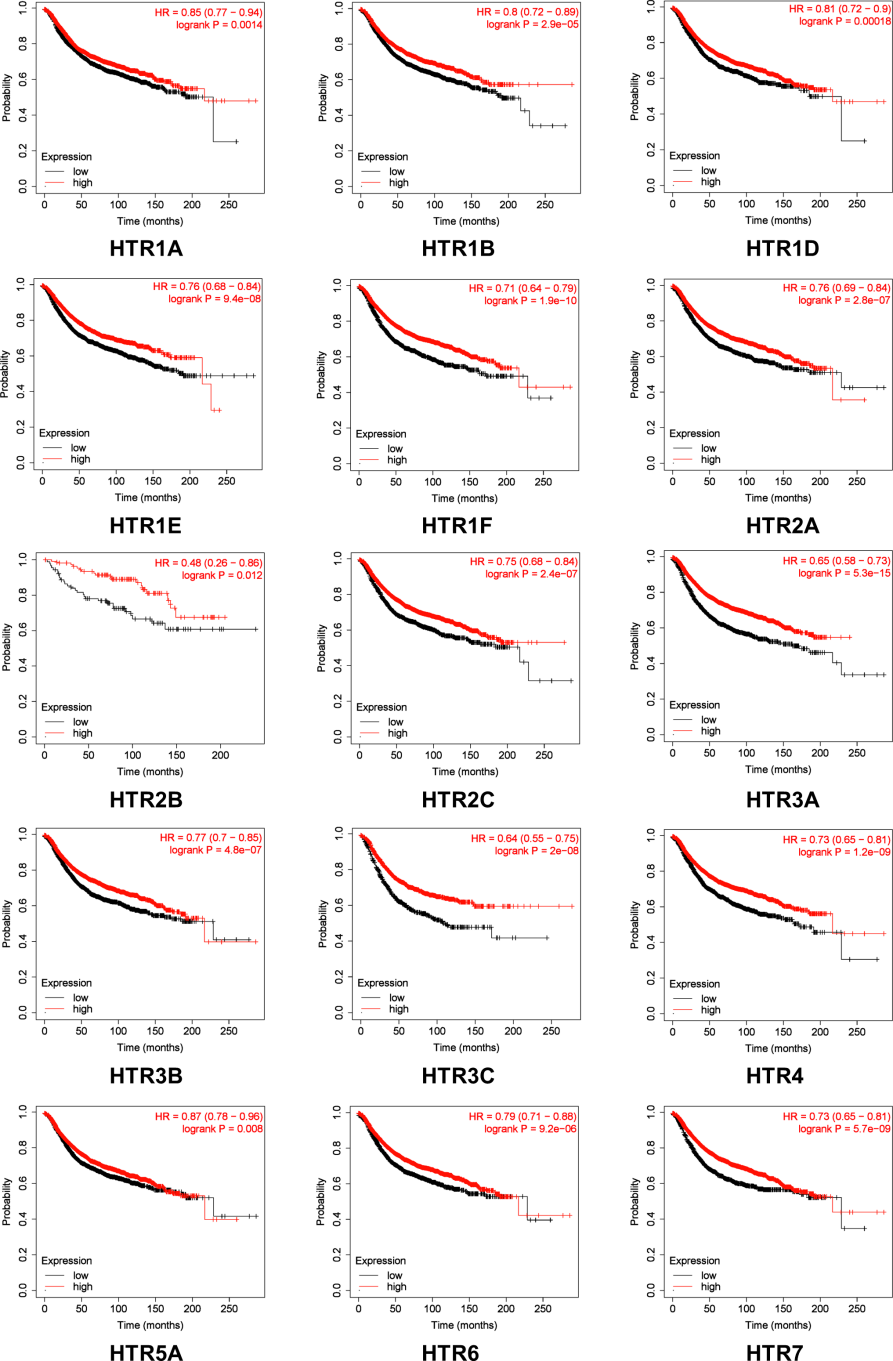
The prognostic value of mRNA Level of HTRs in breast cancer patients analyzed by Kaplan-Meier Plotter. Automatic selection of the optimal cutoff value was used to distinguish between high and low risk patients.

We further evaluated the crucial value of HTRs in predicting the recurrence-free survival (RFS). Kaplan-Meier Plotter tools were applied to evaluate the association between HTRs at different transcription levels and clinical outcomes. The RFS curves are shown in **Figure 3**. Breast cancer patients with higher transcription levels of HTR1A/1B/1D/1E/1F/2A/2B/2C/3A/3B/3C/4/5A/6/7 were significantly associated with longer RFS.

**Figure 4:**
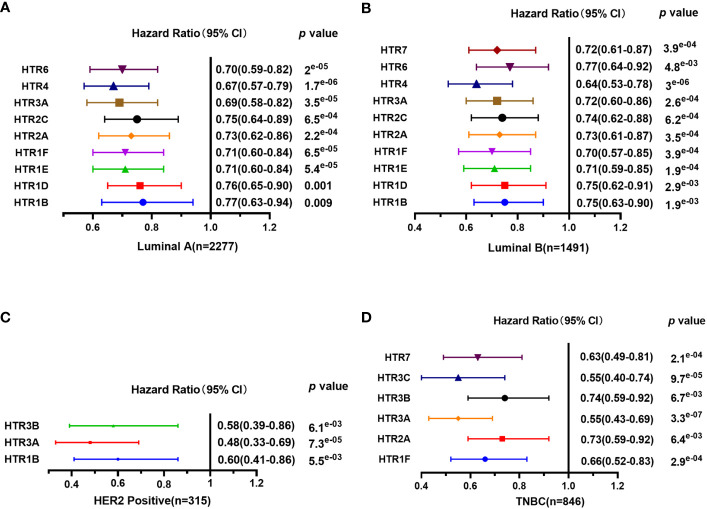
The prognostic value of mRNA Level of HTRs in different breast cancer subtypes analyzed by Kaplan-Meier Plotter. **(A)** Luminal-A breast cancer. **(B)** Luminal-B breast cancer. **(C)** HER2-positive breast cancer. **(D)** TNBC.

Furthermore, Kaplan-Meier Plotter tools were applied to assess the prognostic values of HTRs in different subtypes of in breast cancer (**Figure 4**). The results demonstrated that, consistent with the previous results, patients with high expression panel of HTRs had longer RFS among the four subtypes. In luminal-A breast cancer subtype, the results revealed that the increased levels of HTR1B/1D/1E/1F/2A/2C/3A/4/6 were significantly related to better RFS (*p*< 0.05). Nevertheless, luminal-B breast cancer patients with high HTR1B/1D/1E/1F/2A/2C/3A/4/6/7 expression showed remarkable connection with the improved RFS (*p*< 0.05). Moreover, patients with high HTR1B/3A/3B mRNA levels in HER2-positive breast cancer positively correlated with improved RFS (*p*< 0.05). In contrast, patients with high HTR1F/2A/3A/3B/3C/7 expression in TNBC revealed a significant association with better RFS (*p<* 0.05).

### 3.4 The genetic alteration of HTRs in breast cancer patients and their correlation with prognosis

**Figure 5:**
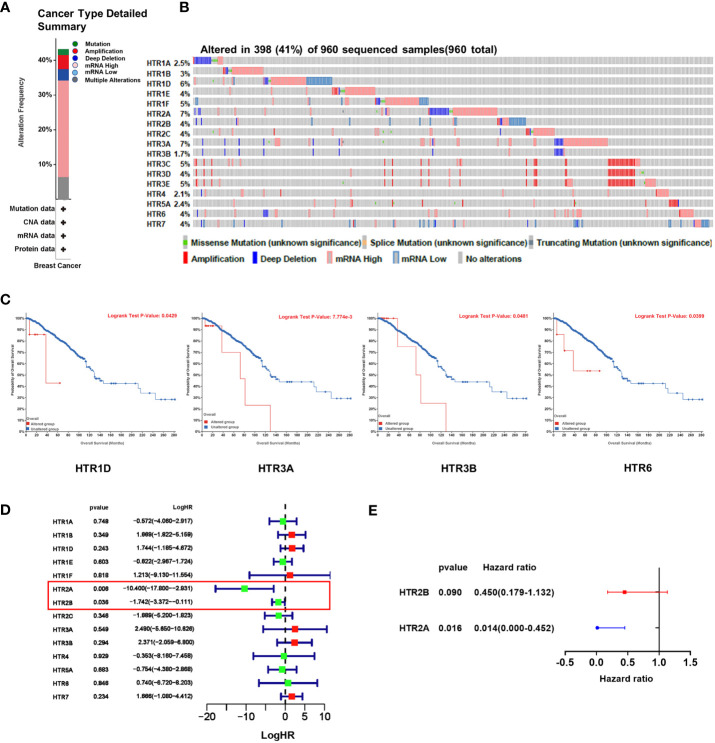
The genetic alteration of HTRs in breast cancer patients and the construction of the risk model based on HTRs from cBioPortal database. **(A)** Summary of alterations in different expressed HTRs in breast cancer patients. **(B)** OncoPrint visual summary of alteration on a query of HTR family members. **(C)** The prognostic value of genetic alteration of HTRs in breast cancer patients. **(D)** Univariate Cox regression analysis of HTRs associated with RFS of breast cancer patients. **(E)** Multivariate Cox regression analysis of HTR2A and HTR2B.

We used the cBioPortal online tool to analyze the genetic alterations of HTRs in breast cancer patients. HTRs were altered in 398 samples out of 960 samples with breast invasive carcinoma (41%), and two or more alterations were detected in 6.4% of 70 cases (**Figures 5A, B**). According to the TCGA dataset, the highest genetic alteration rates in HTRs were HTR2A and HTR3A (7%), the lowest mutation rate was HTR3B (1.7%), and the others were HTR1A (2.5%), HTR1B (3%), HTR1D (6%), HTR1E/2B/2C/3D/6/7 (4%), HTR1F/3C/3E (5%), HTR4 (2.1%) and HTR5A (2.4%) (**Figure 5B**).

To further evaluate the prognostic value of the genetic alteration of HTRs, we explored the association between the altered HTRs and OS by cBioPortal online tool. We found that the genetic alteration of HTR1D, HTR3A, HTR3B, and HTR6 was significantly associated with shorter OS in breast cancer patients (**Figure 5C**), whereas the genetic alteration of other HTRs were not found to be significantly associated with OS (**Supplementary Figure 2**).

### 3.5 Establishment and prognostic value of a 2-gene HTR risk model in predicting breast cancer prognosis

To establish a risk prediction model for predicting RFS in breast cancer patients based on HTR expression levels, we performed a univariate Cox analysis of the 5-HTR genes associated with RFS. The results showed that HTR2A and HTR2B were found significantly related to RFS (**Figure 5D**). Multivariate Cox analysis further identified the 2 HTRs for constructing the risk model (**Figure 5E**). The formula was as follows:


Risk Score=HTR2A×(−4.27)+HTR2B×(−0.8).

**Figure 6:**
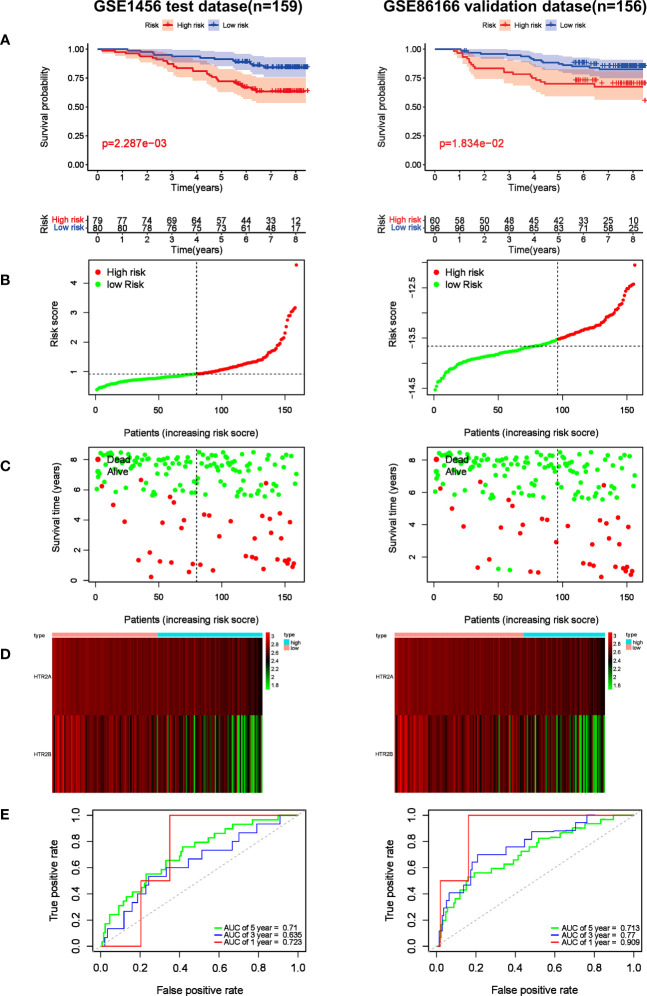
Prognostic effect of the risk signature in breast cancer. **(A)** Kaplan-Meier RFS curves of breast cancer patients in GSE1456 and GSE86166 cohorts. **(B)** The distribution of risk score of breast cancer patients in GSE1456 and GSE86166 cohorts. X‐axis here is patient number and Y‐axis is risk score. **(C)** Survival status of the patients with different risk score in GSE1456 and GSE86166 cohorts. X‐axis is patient number and Y‐axis is survival time. **(D)** A heatmap showing 2 gene expression profiles in GSE1456 and GSE86166 cohorts. **(E)** ROC Analysis of the risk signature in GSE1456 and GSE86166 cohorts.

Based on the formula, the risk scores in the test cohort GSE1456 and the validation cohort GSE86166 were calculated to assess the predictive effect of the model. Subsequently, breast cancer patients were classified into either low or high-risk group by the median value of -13.52. Survival analysis showed that the high-risk patients in the GSE1456 cohort were endowed with a shorter survival time (*p*< 0.01), which was also confirmed by the GSE86166 dataset (*p*< 0.05) (**Figure 6A**). As illustrated in the **Figures 6B, C**, in both GSE1456 and GSE96058 datasets, the higher the risk score, the higher the mortality rate. Heatmap analysis showed that HTR2A and HTR2B were overexpressed in low-risk groups in both GSE86166 and GSE1456 cohorts (**Figure 6D**). In order to test the sensitivity and specificity of the model, the ROC curve was analyzed. As shown in **Figure 6E**, the area under the curve (AUC) was 0.723 and 0.909 at the 1^st^ year in the GSE1456 and GSE86166 cohorts, respectively. Even at the 5^th^ year, the AUC values in both cohorts were still remained above 0.7, indicating the good sensitivity and specificity of the model. The results revealed that the model was effective in predicting the RFS probability.

### 3.6 Effects of HTR2A/2B on the activity of CD8^+^T cells and breast cancer metastasis

**Figure 7:**
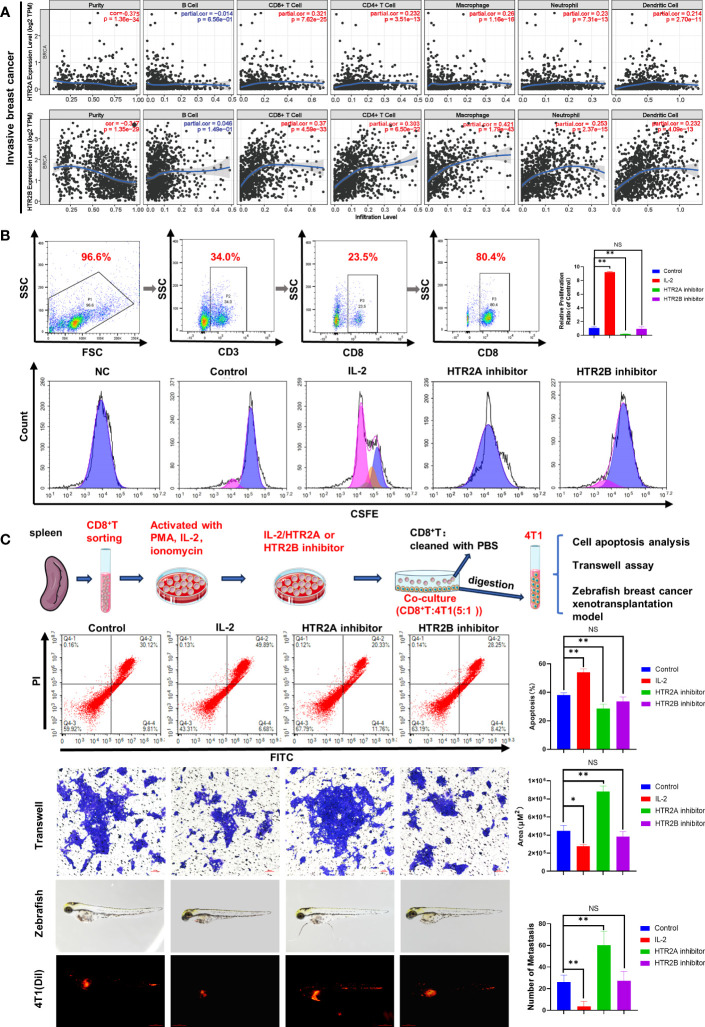
Effects of HTR2A/2B inhibitor on the proliferation and the anti-breast cancer activity of CD8^+^ T cells. **(A)** Correlations between differentially expressed HTRs and immune cell infiltration analyzed by TIMER. **(B)** CD8^+^ T cells were sorted from murine spleens by fluorescence-activated cell sorting technology. The proliferation of CD8^+^ T cells treated with HTR2A/2B inhibitor for 24 h was investigated by CFSE staining assay (n = 3). **(C)** The effects of CD8^+^ T cell pretreated with HTR2A/2B inhibitor on the apoptosis, invasion ability of 4T1 cell (n = 3, scale bar=50μm), and breast cancer zebrafish xenotransplantation model (n = 6, scale bar=0.5mm). Data are represented as the mean ± SD. *p<0.05, **p<0.01. NS, not significant.

To explore the potential mechanisms of HTR2A and HTR2B as prognostic biomarkers for breast cancer, we analyzed the association between these two HTR receptors and immune cell infiltration in the tumor microenvironment through the TIMER database (**Figure 7A**). According to the correlation coefficient, the expression of HTR2A and HTR2B were best associated with the infiltration of CD8^+^ T cells (r = 0.321, 0.37) and macrophages (r = 0.26, 0.421) in invasive breast cancer. Among the different breast cancer subtypes, similar correlations were obtained for the luminal breast cancer subtype but not for HER2-positive breast cancer. In addition, HTR2B expression levels did not correlate with CD8^+^ T cell infiltration levels in TNBC (**Supplementary Figure 3**). It was reported that the presence of tumor-infiltrating lymphocytes (TIL) is related to a favorable outcome in breast cancer patients, thus CD8^+^T cells are considered as a crucial determinant of favorable clinical outcomes (28). As mentioned above, breast cancer patients with poor prognosis have low expression levels of HTR2A and HTR2B and reduced infiltration of CD8^+^ T cells. Meanwhile, it had also shown that T-cell activation leads to the expression of HTR1B, HTR2A and HTR7 (29). Therefore, it was hypothesized that low expression of HTR2A and HTR2B inhibits the activation of CD8^+^ T cells, and subsequently promotes breast cancer invasion and leads to poor prognosis of breast cancer patients.

To test this hypothesis, primary CD8^+^ T cells were isolated from mouse spleen by fluorescence-labeled cell sorting technology, and CD8^+^ T cell proliferation was found to be inhibited by HTR2A inhibitor, detected by CFSE analysis (**Figure 7B**). In order to validate the influence of HTR2A/2B on the invasion ability of breast cancer cells, CD8^+^ T cells pre-treated with HTR2A or HTR2B inhibitors were co-cultured with 4T1 cells and subjected to transwell assay. The results showed that HTR2A inhibitor resulted in the decreased CD8^+^T-induced apoptosis and an enhanced invasion ability of 4T1 cells. However, little effect was observed in the group of HTR2B inhibitor (**Figure 7C**). The zebrafish breast cancer xenografts also revealed that the metastatic ability of 4T1 cells was significantly enhanced after HTR2A inhibitor pre-administration on CD8^+^ T cells. Moreover, these results were also validated in E0771 cells (**Supplementary Figure 4**).

**Figure 8:**
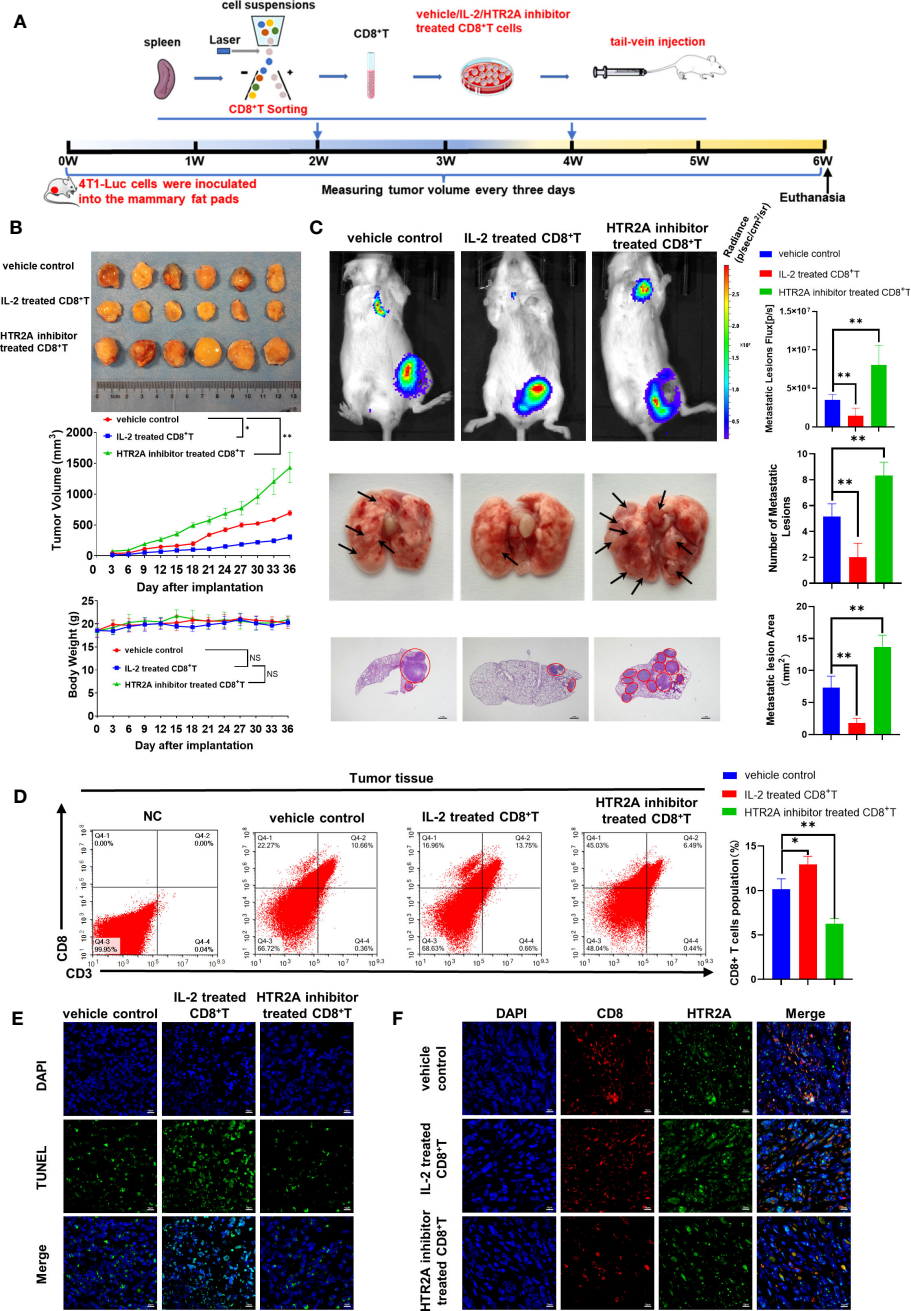
HTR2A inhibitor ketanserin suppresses the proliferation of CD8^+^T cells and promoted breast cancer metastasis. **(A)** The schematic diagram of the animal assay. **(B)** The representative pictures of tumors in each group and tumor growth curves (upper panel) and body weight changes (lower panel) (n=6). **(C)** The lung metastasis of breast cancer xenograft was detected by the *in vivo* luciferase imaging assay (n=6) and HE staining (n=3). The arrows and circles indicate the metastatic tumor foci in murine lungs. Scale bar=1mm. **(D)** The infiltration level of cytotoxic CD8^+^ T cells in breast tumors of each group was evaluated by flow cytometry (n=3). **(E)** Tumor apoptosis in breast cancer was detected by TUNEL assay (n=3). Scale bar=10 μm. **(F)** The infiltration levels of CD8^+^ T cells (red) and the expression level of HTR2A (green) in breast tumor tissues were detected by immunofluorescence assay(n=3). Scale bar=10 μm. Data are represented as the mean ± SD. *p<0.05, **p<0.01.

Finally, *in vivo* studies were conducted to verify that HTR2A suppression inhibits the activation of CD8^+^ T cells and subsequently promotes the growth and metastasis of breast cancer. First, we established a 4T1-Luc breast cancer allograft model by implanting 4T1-Luc cells into the mammary fat pad of Balb/c mice. The pretreated CD8^+^ T cells were given through the tail vein, as indicated in **Figure 8A**. Tumor volume and mice body weight measurements showed that HTR2A inhibitor-treated CD8^+^ T cells significantly promoted breast cancer growth in mice and had little effect on mouse body weight compared to the vehicle control group (**Figure 8B**). In addition, the *in vivo* luciferase imaging assay, gross observation, and HE staining of lung tissue showed that HTR2A inhibitor facilitated breast cancer lung metastasis (**Figure 8C**). To investigate the effect of HTR2A inhibitor on changes of CD8^+^ T composition in the tumor microenvironment (TME), flow cytometry results showed that HTR2A inhibitor treatment significantly reduced the infiltration of cytotoxic CD8^+^ T cells in the TME (**Figure 8D**). In addition, TUNEL staining also confirmed that HTR2A inhibitor reduced the apoptosis of tumor cells (**Figure 8E**). Besides, HTR2A inhibitor significantly suppressed the expression level of HTR2A on CD8^+^ T cells in tumor tissues (**Figure 8F**). Taken together, these findings suggest that HTR2A inhibition could suppress the cytotoxic killing effects of CD8^+^ T cells and result in cancer metastasis. Therefore, HTR2A activation may become a novel therapeutic strategy to prevent and treat cancer metastasis.

## 4 Discussion

As a common comorbidity of breast cancer, depression has a significant physical and psychological impact on the prognosis of breast cancer patients. The role of serotonin in modulating depression was validated in the early 1970s (30). Liu (31) et al. showed that HTR1A overexpression inhibited the proliferation and metastasis of breast cancer cells both *in vivo* and *in vitro*. It has also been shown that HTR1B/1D agonists could induce breast cancer cell death (32). The above studies have shown that HTRs levels in tumors play crucial roles in cancer progression, but 5-HTRs expression in breast cancer varied between studies. For example, Kopparapu PK (33) et al. found that 5-HTR1A expression was strongly observed in breast cancer cell membranes, but was only detected in the cytoplasm of non-malignant cells. 5-HTR1B and 5-HTR2B were mainly expressed in the cytoplasm of breast cancer cells, while 5-HTR4 was only expressed in the nucleus of malignant and non-malignant cell. Pai VP (34) et al. found that HTR1E/1F/2C were upregulated in MCF7 breast cancer cells compared to those in human hypothalamus, while HTR3A was markedly downregulated in all of the breast cancer cell lines. Similar to above studies, our study showed that 5-HTRs were expressed at different levels in different breast cancer types. However, when compared to normal breast tissue, HTR1A, HTR1B, HTR2A, HTR2B, HTR2C, HTR4, and HTR7 were suppressed in high malignancy breast cancer types, whereas they were highly expressed in low malignant breast cancer. These results provide the possibility of applying 5-HTRs as a prognostic indicator for breast cancer.

Gautam J (35) et al. found that serotonin promotes TNBC proliferation and invasion through HTR7 receptor. However, selective serotonin reuptake inhibitors (SSRI) or tricyclic antidepressants (TCA) showed no increased risk for breast cancer concurrence (36). The contradiction between *in vitro* and *in vivo* findings drive us to study the problem from the perspective of bioinformatic analysis. Interestingly, we found that lower expression of 5-HTRs was significantly related to short RFS in all breast cancer patients with more than 200 months of follow-up period, except for HTR5. A recent study reported that HTR1A inhibited the progression of triple-negative breast cancer *via* TGF-β canonical and noncanonical pathways and HTR1A agonists significantly improved patient survival (31). These results indicated that 5-HT and their 5-HTRs may influence breast cancer prognosis through para-cancer pathways instead of direct modulation on cancer cells. In addition, we analyzed the mutation status of 5-HTR genes and their relationship with survival probability. The overall mutation rate of 5-HTR gene was 41% and the OS were shorter in those with HTR1D, HTR3A, HTR3B, and HTR6 mutations. Several studies also analyzed the association of HTRs mutations with certain diseases. For example, 5-HT1A knockout resulted in an anxiety phenotype (37, 38) and abnormal gene expression of HTR2B was found to be a marker of uveal melanoma (39). These findings indicate that the mutated HTRs in breast cancer may be developed as potential targets for drug therapy.

To better present the prognostic value of 5-HTRs in breast cancer, a new risk model consisting of HTR2A and HTR2B was established in our study. The risk signature could effectively discriminate breast cancer patients into high-risk or low-risk group and is capable of predicting RFS in both test and validation cohorts. There are numerous risk models developed for predicting breast cancer mortality, recurrence, or both. The most frequently used predictors include nodal status, tumor size, tumor grade, age at diagnosis, and oestrogen receptor status (40). However, few predictive models based on depression-associated genes (DRG) have been established. Wang (41) et al. established a signature consisting of 10-DRG that predicted OS, which performed well in predicting OS, especially for patients with TNBC. However, this model involved many DRGs, making it difficult to determine a precise biomarker that predicts depression in breast cancer. Given that the predictive model established in our study contains only two genes, it is more conducive for subsequent clinical application. These results suggest that this gene signature may be a powerful tool for identifying the risk population in breast cancer patients who may be developed with depression, and therefore early intervention could be provided to avoid disease progression and finally improve prognosis.

The role of serotonin in modulating neuroimmune circuits is an emerging research field. There is growing evidence supporting that a variety of immune cells regulate serotonin production, storage, response and transportation, including T cells, macrophages, mast cells, dendritic cells and platelets (42, 43). Karmakar S (13) et al. found that serotonin signaling stimulated T cell activation and proliferation, promoted dendritic cells (DCs) maturation, supported B cell development, enhanced NK cell cytotoxicity, and stimulated polarization of macrophages toward the M2 phenotype. For example, through HTR2B signaling, serotonin promotes the maturation of immature CD1a human monocyte-derived DCs (moDCs) upon Toll-Like Receptor3 (TLR3) activation, and upregulates the expression of DC maturation markers like CD80, CD83, and CD8 in moDCs. In macrophages, serotonin receptor signaling regulates the transcription of several genes (AP1, c/EBP, and SRF) *via* HTR2B, which contributes to macrophage activation and polarization through phosphorylation of ERK1/2 molecules. Moreover, serotonin encourages the release of IL-2,16 and IFN-γ from T and B cells *via* HTR2A (44). These studies indicate that serotonin receptors play a crucial role in immune cell responses and inflammatory factor release. Similarly, our study showed that the expression of HTR2A/2B may be significantly associated with the infiltration of immune cells such as CD8^+^ T cells and macrophages, and HTR2A inhibition resulted in the suppression of CD8^+^ T cell proliferation and subsequent 4T1 cell metastasis. Although similar effects were not observed following 5-HTR2B inhibition, its influence on cancer cell growth and metastasis might be mediated by other immune cells.

## 5 Conclusion

In summary, our findings demonstrated that HTRs were significantly correlated with breast cancer RFS and OS, further highlighting the importance of depression in determining the prognosis of breast cancer. More importantly, a new prediction mode consisting of HTR2A and HTR2B was established, and its association with immune cell modulation was proved. Further research is warranted to validate the efficacy of the risk signature in prospective clinical trials and explore the potential molecular mechanisms.

## Data availability statement

The original contributions presented in the study are included in the article/**Supplementary Material**. Further inquiries can be directed to the corresponding authors.

## Ethics statement

The animal study was approved by Guangdong Provincial Hospital of Chinese Medicine Animal Ethics Committee. The study was conducted in accordance with the local legislation and institutional requirements.

## Author contributions 

ZW, NW and DZ conceived and designed the study. DZ and XW conducted the bioinformatic analysis and wrote the manuscript. YZ and SW participated in data interpretation and revised the manuscript. BY and BP contributed to data collection and discussion. All authors contributed to the article and approved the submitted version.
