# Supplementary material for: Integrative dissection of 5-hydroxytryptamine receptors-related signature in the prognosis and immune microenvironment of breast cancer
Source: Front Oncol. 2023 Sep 19;13:1147189. doi: 10.3389/fonc.2023.1147189 (PMC10546427; doi:10.3389/fonc.2023.1147189)
Supplement: Supplementary file 1 [file DataSheet_1.docx]

Supplementary Material

Integrative Dissection of 5-hydroxytryptamine Receptors-related Signature in the Prognosis and Immune Microenvironment of Breast Cancer

**Dandan Zhan, Xuan Wang, Yifeng Zheng, Shengqi Wang, Bowen Yang, Bo Pan, Neng Wang, Zhiyu Wang**

**Correspondence:** Zhiyu Wang: [wangzhiyu@gzucm.edu.cn;](mailto:wangzhiyu@gzucm.edu.cn;) Neng Wang：[ellen0000@126.com](mailto:ellen0000@126.com)

# Supplementary Figures


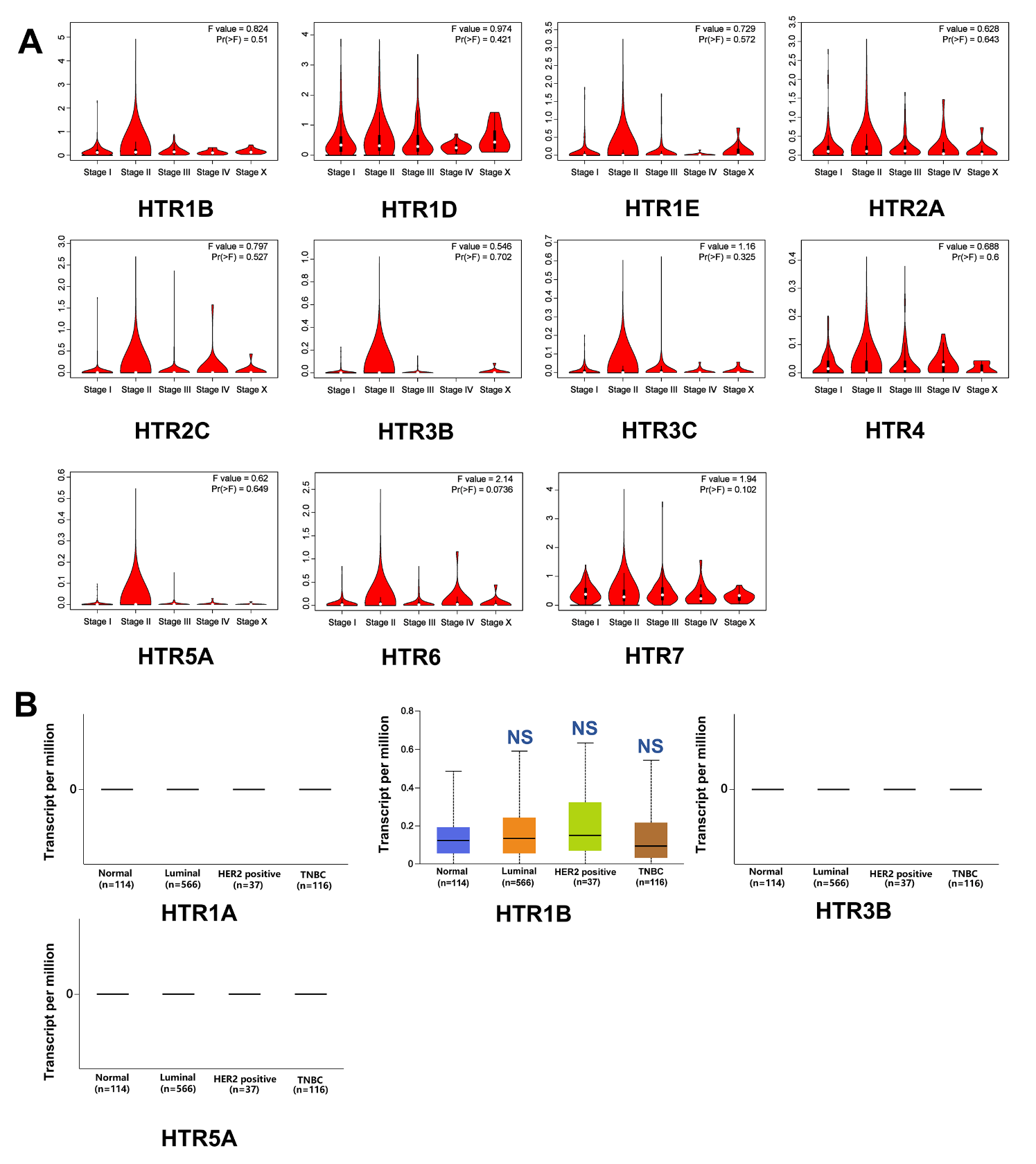


**Supplementary Figure 1.** Relationship between the mRNA Levels of HTRs and the clinicopathological parameters of breast cancer patients. (A) Correlations between HTRs expression and tumor stage in breast cancer patients (GEPIA). (B) Expression of HTRs in different breast cancer subtypes (Online analysis tool: UALCAN). NS, not significant *vs.* the normal group**.**


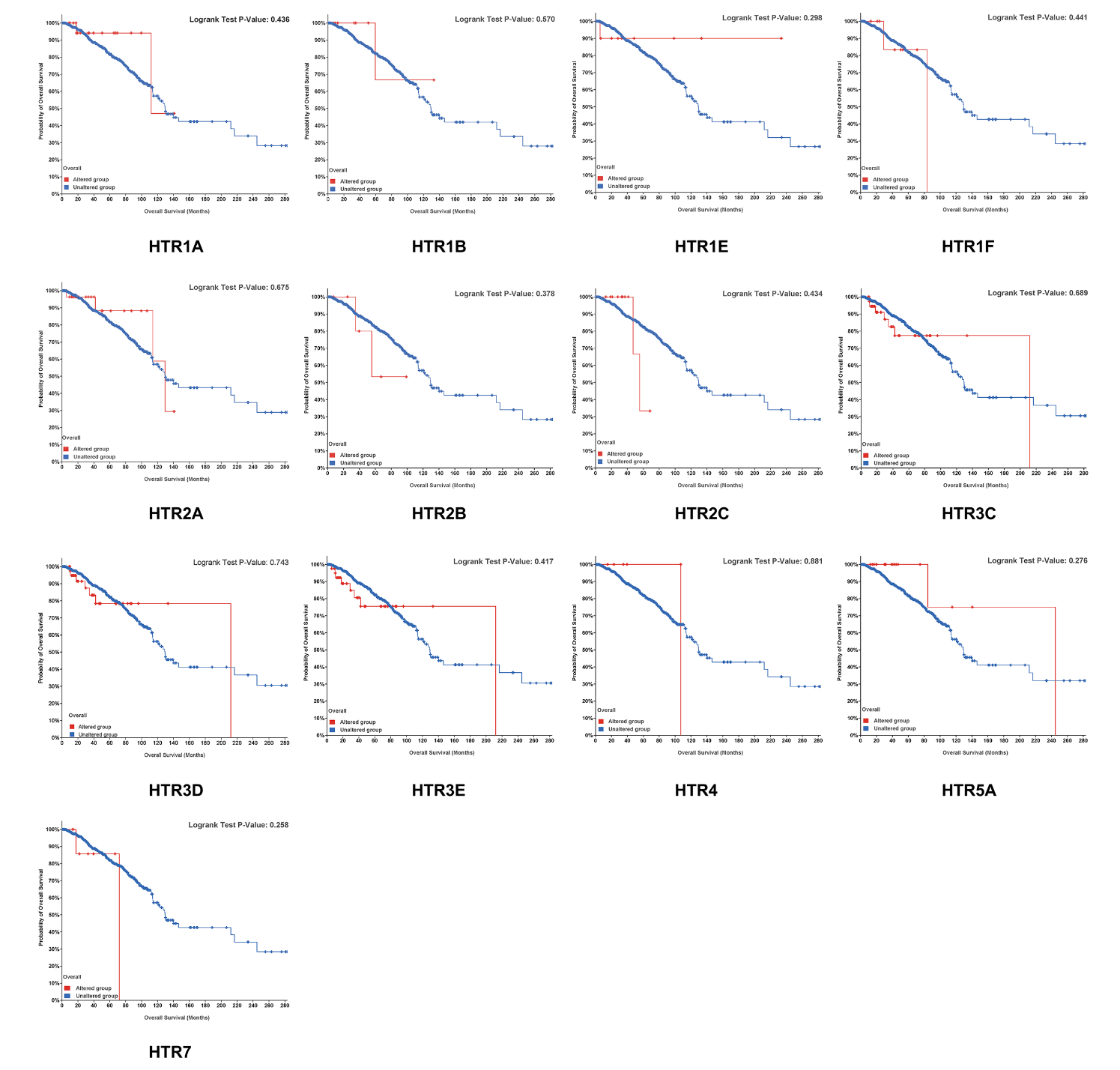


**Supplementary Figure 2.** The prognostic value of genetic alteration of HTRs in breast cancer patients analyzed by cBioPortal.


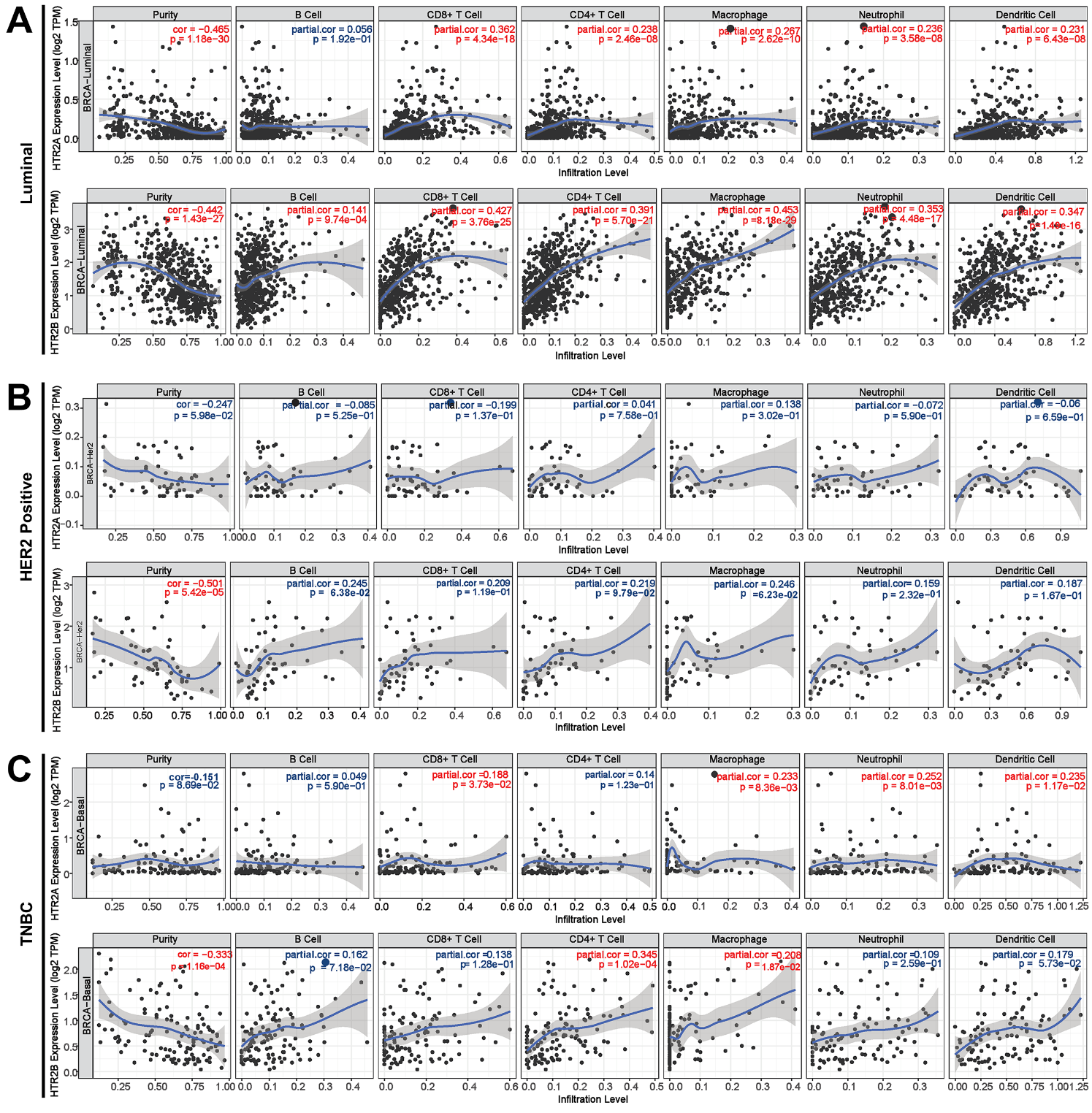


**Supplementary Figure 3.** Correlations between differentially expressed HTRs and immune cell infiltration in different breast cancer subtypes analyzed by TIMER.


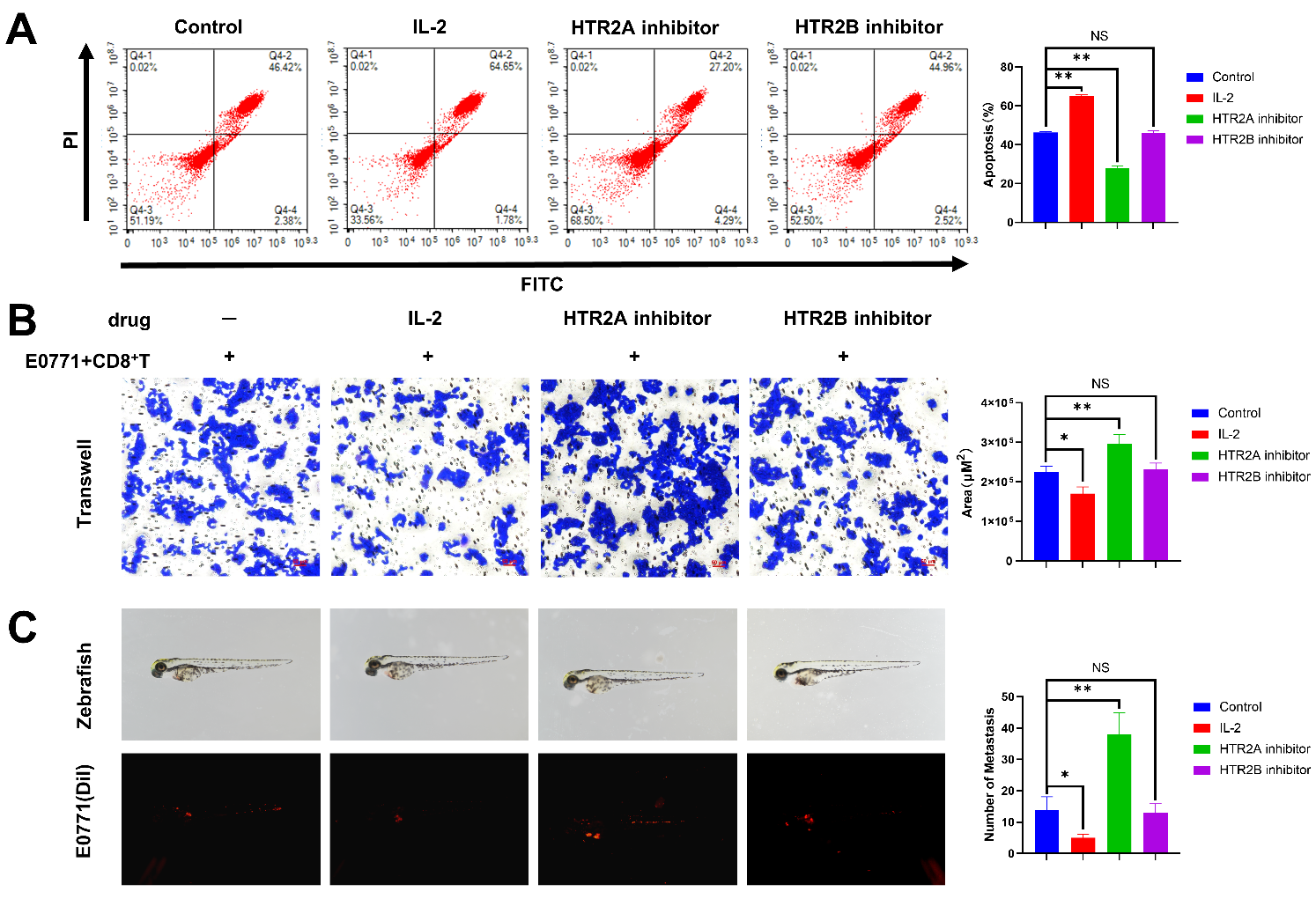


## Supplementary Figure 4. Effect of HTR2A/2B inhibitor on the cytotoxic killing activity of CD8^+^ T cells and breast cancer metastasis. (A) The effects of CD8^+^ T cell pretreated with HTR2A/2B inhibitor on the apoptosis of E0771 cells. (B) The effects of CD8^+^ T cell pretreated with HTR2A/2B inhibitor on the invasion ability of E0771 cells (n = 3). Scale bars represent 50μm. (C) The effects of CD8^+^ T cell pretreated with HTR2A/2B inhibitor on breast cancer growth and metastasis in zebrafish xenografts (n = 6). Scale bars represent 0.5mm. Data are represented as the mean ± SD. **p*<0.05，***p*<0.01.

# Supplementary Table

**Supplementary Table 1. 5-HTR Expression at Transcriptional Level between Different Types of**

**Breast Cancer and Normal Breast Tissues (Oncomine Database)**

|  |  | Type of Breast Cancer versus Normal Breast Tissue |  | Fold Change |  | p Value |  | t Test |  | Source and/or Reference |
| --- | --- | --- | --- | --- | --- | --- | --- | --- | --- | --- |
| 5-HT1AR |  | Invasive lobular breast carcinoma |  | 4.042 |  | 0.007 |  | 3.572 |  |  |
|  |  | Invasive breast carcinoma Stroma |  | 2.266 |  | 6.32E-12 |  | 10.74 |  | Finak Breast Statistics [1] |
|  |  | Invasive breast carcinoma |  | -4.533 |  | 0.0000201 |  | -15.922 |  | Glück Breast Statistics [2] |
| 5-HT1BR |  | Invasive lobular breast carcinoma |  | 2.181 |  | 0.044 |  | 1.979 |  | Turashvili Breast Statistics [3] |
|  |  | Invasive Mixed Breast Carcinoma |  | -8.922 |  | 0.033 |  | -3.597 |  | Radvanyi Breast Statistics [4] |
|  |  | Invasive Ductal Breast Carcinoma |  | -4.248 |  | 0.039 |  | -2.321 |  | Radvanyi Breast Statistics [4] |
| 5-HT1DR |  | Intraductal Cribriform Breast Adenocarcinoma |  | 2.373 |  | 0.03 |  | 3.616 |  | TCGA |
| 5-HT1ER |  | Mixed Lobular and Ductal Breast Carcinoma |  | 2.082 |  | 0.028 |  | 2.32 |  | TCGA |
| 5-HT1FR |  | NA |  | NA |  | NA |  | NA |  | NA |
| 5-HT2AR |  | Fibroadenoma |  | 2.075 |  | 0.026 |  | 2.611 |  | Sorlie Breast Statistics [5] |
|  |  | Male Breast Carcinoma |  | -3.289 |  | 7.76E-08 |  | -8.921 |  | TCGA |
|  |  | Invasive Breast Carcinoma |  | -3.808 |  | 9.76E-18 |  | -10.12 |  | TCGA |
|  |  | Mixed Lobular and Ductal Breast Carcinoma |  | -3.484 |  | 0.0000237 |  | -5.987 |  | TCGA |
|  |  | Intraductal Cribriform Breast Adenocarcinoma |  | -3.675 |  | 0.003 |  | -6.048 |  | TCGA |
|  |  | Invasive Lobular Breast Carcinoma |  | -3.063 |  | 2.31E-09 |  | -6.516 |  | TCGA |
|  |  | Invasive Ductal Breast Carcinoma |  | -3.963 |  | 2.63E-19 |  | -12.098 |  | TCGA |
|  |  | Invasive Ductal and Lobular Carcinoma |  | -3.972 |  | 0.007 |  | -5.216 |  | TCGA |
|  |  | Mucinous Breast Carcinoma |  | -4.763 |  | 0.02 |  | -3.284 |  | TCGA |
| 5-HT2BR |  | Ductal Breast Carcinoma in Situ Stroma |  | 2.185 |  | 0.017 |  | 2.406 |  | Ma Breast 4 Statistics [6] |
|  |  | Invasive Breast Carcinoma Stroma |  | 3.648 |  | 9.84E-08 |  | 9.62 |  | Finak Breast Statistics [1] |
|  |  | Invasive Ductal Breast Carcinoma |  | -2.567 |  | 0.014 |  | -2.536 |  | Radvanyi Breast Statistics [4] |
|  |  | Intraductal Cribriform Breast Adenocarcinoma |  | -4.579 |  | 0.021 |  | -3.936 |  | TCGA |
|  |  | Mucinous Breast Carcinoma |  | -2.455 |  | 0.044 |  | -2.355 |  | TCGA |
| 5-HT2CR |  | Invasive Lobular Breast Carcinoma |  | 2.517 |  | 0.003 |  | 3.16 |  | Turashvili Breast Statistics [3] |
|  |  | Male Breast Carcinoma |  | -2.041 |  | 0.037 |  | -2.847 |  | TCGA |
| 5-HT3AR |  | NA |  | NA |  | NA |  | NA |  | NA |
| 5-HT3BR |  | NA |  | NA |  | NA |  | NA |  | NA |
| 5-HT3CR |  | NA |  | NA |  | NA |  | NA |  | NA |
| 5-HT3DR |  | NA |  | NA |  | NA |  | NA |  | NA |
|  |  | NA |  | NA |  | NA |  | NA |  | NA |
| 5-HT4R |  | Mucinous Breast Carcinoma |  | -2.645 |  | 0.000569 |  | -5.652 |  | TCGA |
|  |  | Male Breast Carcinoma |  | -2.237 |  | 0.000176 |  | -5.961 |  | TCGA |
|  |  | Invasive Ductal Breast Carcinoma Stroma |  | -6.745 |  | 0.03 |  | -2.185 |  | Karnoub Breast Statistics [7] |
| 5-HT3ER |  | NA |  | NA |  | NA |  | NA |  | NA |
| 5-HT5BR |  | NA |  | NA |  | NA |  | NA |  | NA |
| 5-HT6R |  | NA |  | NA |  | NA |  | NA |  | NA |
| 5-HT7R |  | Invasive Mixed Breast Carcinoma |  | -2.548 |  | 0.018 |  | -2.539 |  | Radvanyi Breast Statistics [4] |

NA, not available; TCGA, The Cancer Genome Atlas.

# References

[1] Finak G, N Bertos, F Pepin, S Sadekova, M Souleimanova, H Zhao, et al. Stromal gene expression predicts clinical outcome in breast cancer. *Nature medicine* (2008) 14(5):518-27. Epub 2008/04/29. doi: 10.1038/nm1764.

[2] Glück S, JS Ross, M Royce, EF McKenna, Jr., CM Perou, E Avisar, et al. TP53 genomics predict higher clinical and pathologic tumor response in operable early-stage breast cancer treated with docetaxel-capecitabine ± trastuzumab. *Breast cancer research and treatment* (2012) 132(3):781-91. Epub 2011/03/05. doi: 10.1007/s10549-011-1412-7.

[3] Turashvili G, M Gonzalez-Loperena, E Brogi, M Dickler, L Norton, M Morrow, et al. The 21-Gene Recurrence Score in Male Breast Cancer. *Annals of surgical oncology* (2018) 25(6):1530-1535. Epub 2018/03/10. doi: 10.1245/s10434-018-6411-z.

[4] Radvanyi LG. Targeting the cancer mutanome of breast cancer. *Nature medicine* (2018) 24(6):703-704. Epub 2018/06/06. doi: 10.1038/s41591-018-0065-z.

[5] Sørlie T, CM Perou, R Tibshirani, T Aas, S Geisler, H Johnsen, et al. Gene expression patterns of breast carcinomas distinguish tumor subclasses with clinical implications. *Proceedings of the National Academy of Sciences of the United States of America* (2001) 98(19):10869-74. Epub 2001/09/13. doi: 10.1073/pnas.191367098.

[6] Ma XJ, R Salunga, JT Tuggle, J Gaudet, E Enright, P McQuary, et al. Gene expression profiles of human breast cancer progression. *Proceedings of the National Academy of Sciences of the United States of America* (2003) 100(10):5974-9. Epub 2003/04/26. doi: 10.1073/pnas.0931261100.

[7] Karnoub AE, AB Dash, AP Vo, A Sullivan, MW Brooks, GW Bell, et al. Mesenchymal stem cells within tumour stroma promote breast cancer metastasis. *Nature* (2007) 449(7162):557-63. Epub 2007/10/05. doi: 10.1038/nature06188.
